# Mental Health and Work Experiences of Interpreters in the Mental Health Care of Refugees: A Systematic Review

**DOI:** 10.3389/fpsyt.2021.710789

**Published:** 2021-10-18

**Authors:** Angelika Geiling, Christine Knaevelsrud, Maria Böttche, Nadine Stammel

**Affiliations:** ^1^Division of Clinical Psychological Intervention, Department of Education and Psychology, Freie Universität Berlin, Berlin, Germany; ^2^Zentrum Überleben, Berlin, Germany

**Keywords:** interpreter, mental health, refugee, trauma, secondary stress, health care, stress

## Abstract

**Background:** Interpreters often play a crucial role in the health care of refugees. Although interpreters working with refugees are regularly confronted with emotionally stressful content, little is known about their work-related stress and psychological well-being. Primarily qualitative studies indicate increased emotional stress in interpreters, and difficulties in handling the traumatic content from their clients. Additionally, the working conditions of interpreters appear to be demanding, due to low payment and a lack of supervision or adequate preparation.

**Objective:** The presented systematic review aimed to identify and summarise quantitative and qualitative research on the mental health of interpreters in the mental health care of refugees.

**Method:** A systematic search was performed in five databases, and specific interpreting journals were searched. After removal of duplicates, 6,920 hits remained. Eligible studies included quantitative, qualitative, and mixed-methods studies as well as case studies and grey literature. The studies aimed to examine mental health aspects or work experiences of spoken language interpreters in mental health care settings for adult refugees.

**Results:** Altogether, 25 studies were identified, including six quantitative and 19 qualitative studies. Studies were analysed and presented narratively. In the analysis of the qualitative studies, three themes emerged: “Emotions, behaviour, and coping strategies,” “Working in a triad,” and “Working environment.” In the quantitative studies, interpreters showed heightened levels of emotional stress and anxiety, and secondary traumatic stress reactions. In several qualitative studies, interpreters described a devaluing health care system and stressful working conditions with a lack of support structures.

**Conclusion:** Overall, the results indicate a high level of stress among interpreters working with refugees. Quantitative data are sparse, and studies employ heterogeneous assessments in diverse study settings. Therefore, future quantitative research is necessary to consistently investigate interpreters' mental health in different mental health care settings.

**Systematic Review Registration:** https://www.crd.york.ac.uk/PROSPERO/, identifier: CRD42019117948.

## Introduction

Interpreters play a central role in providing equal access to health care for refugees and immigrants (1). Without an interpreter, communication may be limited, as refugees and staff members like counsellors, physicians, or civil servants in the resettlement countries in particular often do not speak the same language (2, 3). In addition to translating, interpreters frequently give cultural explanations or advice (4) and are therefore also considered as cultural brokers (5).

The use of interpreters in public service is generally underregulated (6). It appears to be difficult to organise professional interpreters, and clinical staff are often unaware of the importance of professional interpreters. Therefore, family members or friends often serve as *ad hoc* interpreters (1). Moreover, health insurance companies do not generally cover the costs of interpreters (7), even though it is strongly recommended to work with professional or at least trained interpreters (8). Interpreters who are affiliated or registered with a professional interpreting service or institute are sometimes bound by a code of conduct that emphasises, for example, confidentiality, impartiality or the benefits of supervision, as in the “code of ethics” and “code of conduct” of the AUSIT (Australian Institute of Interpreters and Translators) in Australia or the “code of ethics and standards of practice” of the National Council on Interpreting in Health Care Develops National Standards for Interpreters (NCIHC) in the USA. Additionally, several guidelines and policy articles have provided advice and suggestions for work with interpreters in the context of mental and physical health, e.g., clarifying role expectations of the interpreter, and brief feedback meetings before and after consultations/therapy sessions [e.g., (9)]. However, a recent review indicated a lack of support in terms of supervision, training, or preparation for interpreters (10).

Usually, interpreters work in a triadic setting with a client and, for example, a therapist. Role expectations and dynamics regarding the interpreter's role have therefore been discussed with respect to how they affect the interpreter's work experience (4, 5). In their literature review, Sleptsova et al. (5) outlined that each party in the triad expresses different expectations of the interpreter's role: Practitioners often expect interpreters to remain impartial, while clients wish for help and guidance in the health care system. A study addressing interpreters' own perceptions of their roles in health care settings identified several different roles, e.g., functioning as an advocate by empowering the client or being a conduit by only translating (4). Additionally, interpreters are under pressure within their work situation, as mostly, the practitioner decides whether the interpreter will return for another appointment (11), thus creating a power imbalance.

A growing body of qualitative literature indicates physical and mental exhaustion among interpreters in various settings und with different client populations, e.g., refugees, migrants, or clients with limited English proficiency (LEP) in English-speaking countries [e.g., (6, 12–14)]. Besides reported stress reactions, interpreting has been described as meaningful and resourceful with respect to the interpreter's own traumatic experiences, as the interpreters indicated that interpreting for clients with similar helped them to process their own experiences (15). Moreover, compassion satisfaction (CS), which comprises the satisfaction and fulfilment due to work, was found to be significantly higher in an interpreter sample than in other professions (16).

Working with refugee clients stands out from the regular work of an interpreter (2). Many refugees have experienced war- and flight-related traumatic events (17) and show a high prevalence of trauma-related mental disorders such as depression and posttraumatic stress disorder (PTSD) (18). Therefore, interpreters working with refugees are frequently confronted with highly traumatic content (19, 20). As interpreters often translate in the first person, it has been suggested that the effect of the traumatic content is even more stressful for the interpreter (21). Stress reactions as a consequence of another person's trauma are often referred to as secondary traumatic stress (STS), vicarious traumatization (VT), or compassion fatigue [CF, i.e., STS and burnout, (22)]. These constructs have been frequently investigated in helper populations in which practitioners are indirectly confronted with the trauma of another person, e.g., trauma therapists or social workers (23, 24). Therefore, Mehus and Becher (16) investigated STS in interpreters in the USA, who had heard traumatic content as part of their work, and found significantly higher levels of STS compared to other professions.

In the therapeutic context, language is key to facilitating treatment (25), and some skills are particularly useful for interpreters (1), e.g., knowledge of psychopathology as well as correct terminology. Two recent studies addressed difficulties for interpreters specific to the mental health setting (11, 20), and emphasised that the mental health care structures require complex roles and emotionally demanding skills.

So far, several studies have investigated interpreters' experiences regarding patients with general limited English proficiency (LEP) [e.g., (4, 16)]. However, little is known about the mental health of interpreters in the mental health care of refugees. To the best of our knowledge, only one meta-synthesis has investigated interpreters' experiences in health and mental health care settings, and this was based exclusively on qualitative studies (10). A scoping review also synthesised research on challenges and opportunities in interpreter-assisted mental health setting with refugee clients (26). Therefore, the aim of this review is to systematically summarise and report the mental health and work experiences of spoken language interpreters in both qualitative and quantitative studies in order to gain a better and more comprehensive understanding of stress reactions and potentially associated risk and protective factors due to interpreting for refugee clients.

## Methods

The review was registered at PROSPERO (CRD42019117948) and is reported in accordance with the PRISMA statement (27).

### Search Strategy and Inclusion Criteria

The following inclusion criteria for eligible studies were defined according to the PI(E)CO schema (28): Population–paid spoken language interpreters for adult refugee clients; Exposure–mental health setting (e.g., counselling or therapy) as one of the interpreters' mentioned work settings; Comparison–(no) comparison group was investigated; Outcome–interpreters' mental health and/or work experiences reported by interpreters themselves (not by a third person). As recent research indicated that there might be a difference in psychological strain between paid and voluntary interpreters (29), the present review focussed on studies in which paid interpreters participated. Studies were included if mental health was mentioned as one of the work settings. Accordingly, exclusion criteria regarding the population of interpreters were as follows: (1) interpreters for children and adolescents only, (2) interpreters for any form of sign language only, (3) interpreters working on a volunteer basis only and therefore not paid, (4) sample did not include interpreters working in a mental health setting, (5) client population did not include refugees or asylum seekers, (6) children and/or adolescents working as interpreters. Articles had to be written in the English or German language and to report qualitative, quantitative or mixed-methods studies. Journal articles, book chapters, and dissertations were included, whereas reviews and Master and Bachelor theses were excluded.

### Study Selection

Articles were retrieved between the 5th and 7th November 2018. The search was updated on the 1st September 2020. The English search terms were: interpreter^*^ OR translat^*^ AND stress^*^ OR trauma^*^ OR mental health OR health care OR work^*^ OR experience^*^ AND refugee^*^ OR asylum seeker^*^ OR survivor^*^ OR migrant^*^ OR immigrant^*^ OR limited english proficien^*^ OR migration^*^ OR immigration^*^. Six databases were searched (PsycINFO, PsycARTICLES, Web of Science, PubMed, PSYNDEX, and Proquest). No time frame for publication date was applied. After the screening of eligible studies, further literature was identified by snowballing. Additionally, a backward citation search was conducted based on all included studies. Furthermore, publications of the Critical Link Conference and of the International Journal of Interpreter Education were searched. Two independent researchers screened titles/abstracts and full texts (first rater: AG, second rater: initial search: TW/updated search: VB). Interrater reliability was calculated using Cohen's kappa (30). In all screening stages, any disagreements were resolved by discussion.

### Risk of Bias

Two independent researchers [first rater: AG (100%), second rater: TW (88%)/CM (12%)] rated five items (items 2, 4, 6, 8, and 9) of the CASP (31) for qualitative studies and the Mixed Methods Appraisal Tool (MMAT) for quantitative studies (32). Possible responses were “yes,” “no,” or “can't tell.”

### Data Extraction

Two piloted codebooks for data extraction for qualitative and for quantitative studies were written and discussed with a second researcher (NS). Sample characteristics and study design for both types of studies were extracted by one researcher (AG) and 50% randomly chosen studies were double-checked by a second researcher (JK). For qualitative studies, the complete results sections of every qualitative study were first extracted, and 50% of the studies were then screened for relevant parts independently by two researchers (AG, MM). All results of quantitative studies were extracted by one researcher (AG).

### Synthesis

#### Qualitative

**Figure 1:**
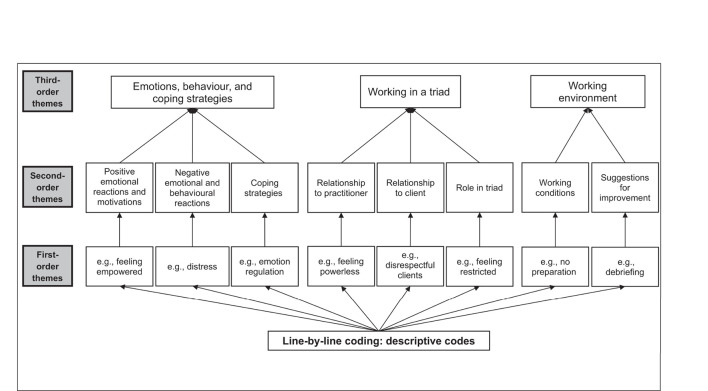
Hierarchical structure of first-, second-, and third-order themes. First-order themes were numerous (*n* = 88) and are only shown as examples.

The coding for qualitative studies was processed in three steps based on principles of thematic analysis (33, 34). The themes were structured into first-order, second-order, and third-order themes. First-order themes emerged as descriptive and interpretative themes from the codes after the line-by-line coding. The descriptive themes were clustered into second-order themes. Lastly, the third-order themes, at the top of the hierarchical thematic structure, summarised the first- and second-order themes (Figure 1). Two independent researchers (AG, MM) coded around 50% of the studies during the line-by-line coding and clustered 20% randomly chosen codes into first-order themes. The remaining codes were clustered by one researcher (AG), and unclear codes were discussed with an independent researcher (JK). The computer software MAXQDA 2018 [VERBI (35)] was used for qualitative coding.

#### Quantitative

It was not possible to carry out a meta-analysis because too few quantitative studies were available. Therefore, study characteristics and key findings are presented.

#### Overall Synthesis

The results of qualitative and quantitative studies are reported narratively according to the qualitative thematic structure and complemented with thematically corresponding quantitative key findings.

## Results

### Included Studies and Study Characteristics

**Figure 2:**
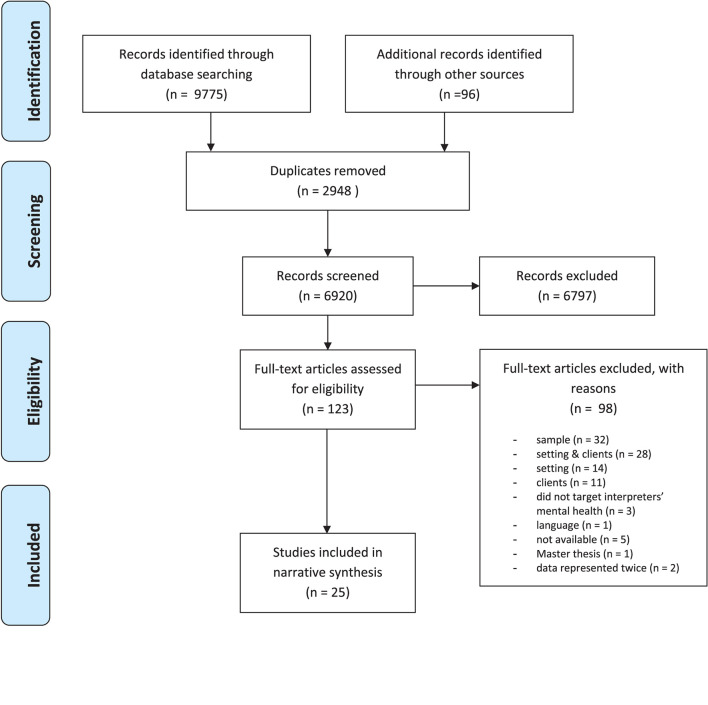
Flowchart of study identification and selection according to the PRISMA flow diagram (27).

In total, 25 studies (19 qualitative and 6 quantitative) were included. The flow chart of included and excluded studies is depicted in Figure 2. Interrater reliability was calculated for the full-text screening using Cohen's Kappa for categorical variables (30), and lay at κ = 0.86, showing good agreement. Two studies were represented twice, and the article with the most comprehensive information was chosen in each case (36, 37). Sample and study characteristics are displayed in Table 1. The eligible studies also included five dissertations (42, 48, 49, 51, 55). Sample sizes varied between *n* = 3 and *n* = 90.

**Table 1:** Sample and study characteristics.

**References**	**Study location**	**Sample size**	**Recruitment location**	**Method for data collection**	**Gender: female (%)**	**Age (mean)**	**Interpreting experience (mean in years)**	**Education as interpreter (%)**	**Receiving supervision (%)**	**Own flight experience (%)**	**Own trauma experience**
**Qualitative studies**											
Butler (38)	England	3	Health service	Interview	100%	NA	NA	NA	NA	NA	NA
Celik and Cheesman (39)	Wales	25	Mixed^a^	Semi-structured interview	NA	NA	NA	20%	100%	NA	NA
Crezee et al. (36)	New Zealand	90	Interpreting service/agency	Online survey, focus group	75.6%	NA	NA	19.6%	NA	22%	NA
D'Ardenne et al. (40)	England	3	Interpreting service/agency^b^	Focus group	NA	NA	NA	100%	NA	NA	NA
Doherty et al. (41)	Scotland	18	Interpreting service/agency^b^	Online survey	NA	NA	6.1	NA	NA	NA	NA
Dubus (2)	USA	36	Interpreting service/agency	Semi-structured interview	50%	NA	NA	NA	NA	NA	NA
Grant (42)	Canada	4	Mixed^a^, ^b^	Interview; focus group	100%	NA	NA	100%	NA	25%	NA
Green et al. (43)	England	6	Mixed^a^	Semi-structured interview	33%	40.5	11.33	NA	NA	100%	NA
Holmgren et al. (12)	Denmark	12	Humanitarian/refugee care organisation	Semi-structured interview	66%	30	NA	NA	NA	91%	NA
Lipton et al. (44)	Australia	15	Interpreting service/agency^b^	Ethnographic interview	NA	NA	NA	NA	NA	NA	NA
Miller et al. (45)	USA	15	Humanitarian/refugee care organisation	Semi-structured interview	NA	NA	NA	NA	NA	NA	NA
Mirdal et al. (46)	Denmark	8	Humanitarian/refugee care organisation	Semi-structured interview	75%	NA	NA	100%	100%	NA	NA
Mirza et al. (47)	USA	5	Humanitarian/refugee care organisation	Interview	20%	NA	4.2	60%	NA	NA	NA
Myler (48)	England	8	Interpreting service/agency	Semi-structured interview	75%	42.6	8.69	37.5%	NA	NA	NA
Resera et al. (20)	England	12	Humanitarian/refugee care organisation^b^	Focus group	83%	NA	NA	42%	NA	NA	NA
Robertson (49)	England	3	Health service	Semi-structured interview	NA	NA	NA	0%	NA	NA	NA
Splevins et al. (50)	USA	8	Humanitarian/refugee care organisation	Semi-structured interview	75%	46	NA	NA	NA	NA	100%
Williams (51)	USA	9	Interpreting service/agency	Semi-structured interview	44%	NA	3.1	22%	NA	100%	NA
Williams (52)	England	8	Health service	Semi-structured interview	NA	NA	NA	NA	NA	NA	NA
Quantitative studies											
Birck (53)	Germany	5	Humanitarian/refugee care organisation	Self-report questionnaire	NA	NA	NA	NA	NA	NA	NA
Denkinger et al. (54)	Germany	11	Humanitarian/refugee care organisation	Self-report questionnaire	NA	NA	NA	NA	NA	45.5%	27.3%
Kindermann et al. (29)	Germany	64	Mixed^a^	Self-report questionnaire	56.2%	37.3	3.3	NA	23%	25.5%	58%
Shlesinger (55)	USA	52	Humanitarian/refugee care organisation^b^	Self-report questionnaire	71.1%	40.1	NA	NA	40%	NA	51%
Teegen and Gönnenwein (56)	Germany	51	Humanitarian/refugee care organisations	Self-report questionnaire	73%	35	4	NA	41%	NA	90%
Wichmann et al. (37)	Germany	60	Mixed^a^	Self-report questionnaire	73%	NA	NA	55%	25%	33%	50%

a *Various recruitment locations, e.g., hospitals, services, charities.*

b *Mixed client population: refugees/asylum seekers and other clients (e.g., migrants, LEP clients), NA, not available*.

The studies were carried out in Canada (*k* = 1), USA (*k* = 5), Australia (*k* = 1), New Zealand (*k* = 1), England (*k* = 8), Scotland (*k* = 1), Wales (*k* = 1), Denmark (*k* = 2), and Germany (*k* = 5). Seven studies did not focus specifically on interpreters and also included other professions, such as caregivers, therapists, or administration staff (40, 45–47, 49, 53, 54). Regardless of profession, the third person besides the refugee client and the interpreter will henceforth be referred to as the practitioner, denoting any person who carries out any kind of care for refugees, e.g., a therapist, a civil servant, or a counsellor.

Eight studies were conducted in a mixed setting describing interpreters' experiences in the mental health setting and other settings [e.g., general internal medicine or court, (2, 12, 29, 36, 37, 52, 54, 56)]. In one study, 44% of the interpreters were paid whereas the rest worked voluntarily (29). Three studies did not indicate how many interpreters were paid (39, 52, 55). In one study, 3% of the sample were sign language interpreters (37). One quantitative study investigated several measurements of mental health and will therefore be presented in more detail (29).

### Overall Synthesis

Three superordinate third-order themes emerged, which summarise the first- and second-order themes in the thematic analysis of qualitative studies: (1) “Emotions, behaviour, and coping strategies,” (2) “Working in a triad,” and (3) “Working environment.” These are split into eight second-order themes (negative emotional and behavioural reactions, positive emotional reactions and motivations, coping strategies, relationship to client, relationship to practitioner, role in triad, working conditions, suggestions for improvement of the work as an interpreter) and 88 first-order themes. The third-order theme “Emotions, behaviour, and coping strategies” describes reactions and coping behaviour of interpreters as a consequence of their job, whereas the third-order themes “Working in a triad” and “Working environment” primarily include work experiences and their potential impact on interpreters' mental health and job satisfaction. Every second-order theme is first described with selected examples of first-order themes, and then supplemented with the corresponding results of the quantitative studies. Table 2 provides an overview of the contribution of quantitative study results to the second- and third-order themes. Additionally, an overview of the questionnaires used in the quantitative studies can be found in the Supplementary Material.

**Table 2:** Contribution of quantitative studies to second- and third-order themes of the thematic analysis.

**References**	**Emotions, behaviour, and coping strategies**			**Working in a triad**			**Working environment**	
	*Positive emotional reactions and motivations*	*Negative emotional and behavioural reactions*	*Coping strategies*	*Relationship to practitioner*	*Relationship to client*	*Role in triad*	*Working conditions*	*Suggestions for improvement*
Birck (53)	X	X						
Denkinger et al. (54)		X						
Kindermann et al. (29)		X	X				X	X
Shlesinger (55)	X	X						
Teegen and Gönnenwein (56)		X	X					
Wichmann et al. (37)	X	X	X	X	X	X	X	X

*Second-order themes in italic, third-order themes in bold*.

#### Emotions, Behaviour, and Coping Strategies

The third-order theme “Emotions, behaviour, and coping strategies” comprises three second-order themes (negative emotional and behavioural reactions, positive emotional reactions and motivations, and coping strategies) and 39 first-order themes which emerged from the qualitative studies (Table 3). The first third-order theme comprises all emotional reactions identified in quantitative and qualitative studies, as well as reported coping mechanisms as a consequence of the work as an interpreter.

**Table 3:** Emotions, behaviour, and coping strategies as third-order theme specified by first-order and second-order themes.

**Second-order theme**	**First-order theme**	**References**
Negative emotional and behavioural reactions	Sadness and crying	(12, 36, 38–41, 43, 45, 48, 50, 51)
	Helplessness	(12, 36, 50)
	Hopelessness	(41, 50)
	Powerlessness	(39, 41, 51)
	feeling useless	(41, 51)
	social withdrawal	(12, 41)
	feeling torn	(2, 20)
	Fear	(12, 40, 45, 50–52)
	Guilt	(12, 41)
	Exhaustion	(12, 40, 41, 44, 45, 48)
	Distress	(2, 12, 36, 40, 44, 50)
	Anger	(38, 41, 48, 50, 51)
	Struggle in handling own emotions	(12, 20, 36, 40, 41, 43, 48, 51)
	Hyperarousal	(12, 44, 45, 51)
	Experiencing intrusions	(2, 12, 39, 43, 45)
	Experiencing physical reactions	(12, 45, 50)
	Lack of job satisfaction	(12, 44)
	Emotional reactions over time	(39, 41, 45, 50)
	Traumatic content stressful	(2, 20, 36, 38–41, 43–45, 50)
	Developing negative perceptions of world and self	(38, 43, 44, 50, 51)
	Difficult to be detached	(12, 39–41, 51)
Positive emotional reactions and motivations	Feeling empowered	(42, 51)
	Satisfying job	(39, 41, 42, 44, 45, 48, 51)
	Motives to be an interpreter	(2, 12, 39, 43, 44, 48, 51, 52)
	Seeing client's recovery as satisfying	(20, 39, 41, 50)
	Personal growth	(39, 41–43, 45, 50)
	Developing positive world perceptions	(39, 50)
	Self-healing through interpreting	(43–45, 48, 50, 51)
Coping strategies	Distracting activities	(12, 36, 41, 48, 50)
	Interpreting is just a job	(40, 41, 48, 49)
	Cognitive strategies	(12, 36, 38, 39, 41, 43, 45, 49, 51)
	Emotion regulation	(12, 20, 36, 48, 50, 51)
	Religious practises	(41, 50)
	Social support	(12, 38, 41, 43, 44)
	Maladaptive or lack of coping strategies	(12, 36, 41, 44)
	Limiting or quitting work	(12, 36, 40, 41, 44, 51)
	Separation of private and work life	(39, 41, 48, 51)
	Support by staff	(41, 43, 44, 48, 50)
	Training and work experience help	(41, 48, 50, 51)

##### Negative Emotional and Behavioural Reactions

Interpreters reported specific negative emotions related to the interpreting, e.g., distress (*k* = 6), hyperarousal (*k* = 4), physical exhaustion (*k* = 6), or feeling anxious (*k* = 6). Across all qualitative studies, sadness and crying (*k* = 11) were the most frequently mentioned reactions. Interpreters reported several reasons for feeling sad, e.g., because of the client's story (38, 50) or when stories were perceived to be similar to their own experiences (41, 51). For some interpreters, the traumatic content was associated with feelings such as shock (39, 41, 50) or disbelief (39, 50). Regarding psychological stress over time, some interpreters reported that they experienced an initial peak when they first began their job as interpreters (45). Sometimes, the stress decreased after a time (45, 50, 51), or interpreters became accustomed to it (50) and showed no long-term mental health effects (45). Particularly when coping strategies were acquired (50), feelings of stress seemed to change into a sense of fulfilment and work pleasure (39, 50).

A German quantitative study investigated stress, depression, and anxiety symptoms in voluntary and paid interpreters (29). Interpreters showed significantly higher stress and anxiety symptoms than in representative population samples, and depressive symptoms indicated a non-significant trend towards higher symptom levels than in a representative population. Clinically relevant anxiety symptoms were found in 16.1% of the sample, and 8% showed moderate to severe depressive symptoms. Voluntary interpreters had significantly higher levels of depressive symptoms than did paid interpreters, whereas no differences were found for stress and anxiety. *The authors suggested that voluntary interpreters probably would receive no informal debriefings or adequate trainings as their paid colleagues and may therefore have reported higher depression levels than paid interpreters* (29). Female interpreters reported significantly higher symptom levels for stress, anxiety, and depression than did male interpreters. No significant associations were found regarding interpreters' own flight and work experiences. Additionally, stress, depression, and anxiety were significantly negatively correlated with a secure attachment style, whereas anxiety was positively associated with a dismissing and preoccupied attachment style. However, significant negative correlations emerged between psychological strain (stress, anxiety, depression) and social support and sense of coherence. In another German study, interpreters showed significantly lower levels of depressive symptoms compared to the normal population (56). The authors suggested that interpreters might have downplayed distress in general, as 20% of the sample completing the depression questionnaire had to be excluded from the analysis of depressive symptoms based on the lie criterion.

Two studies reported on the prevalence of PTSD in interpreters, which ranged from 9 to 10% (29, 56). In one study, interpreters who had a refugee background had significantly higher PTSD symptoms than those who did not (29). The higher PTSD symptoms could therefore be caused by their own traumatic experiences. Another study compared interpreters with and without (partial) PTSD (56). Partial PTSD was assumed if the criteria A1, A2 were fulfilled as well as 2 criteria of B-D and full PTSD if criteria A1, A1, and B-D were fulfilled according to the DSM-IV. Participants with (partial) PTSD in the study of Teegen and Gönnenwein (56) reported significantly more stress because of interpreted traumatic content, had higher depressive symptoms, and significantly more chronic diseases (e.g., allergies, migraine, tinnitus). Interpreters with partial or full PTSD also reported seeking more professional help and speaking less often with their partners about their work compared to interpreters without a (partial) PTSD. Additionally, PTSD symptoms in the same study were generally significantly positively associated with depression, emotional communication ability, and traumatic content at work. PTSD was not significantly related to the use of supervision. In addition, 80% of the total sample felt fear, helplessness, and terror while interpreting traumatic content. Several situations were mentioned as especially stressful: interpreting rape, interpreting for emotionally devastated clients, contact with clients who wanted to let out their sadness on the interpreter, and coping with clients' statements which bore serious consequences for the clients (56).

STS was the most frequently investigated construct in quantitative studies, and was assessed using the same questionnaire [Questionnaire for Secondary Traumatization, FST (57)] in three studies. Between 12 and 17% of the interpreters had experienced moderate STS and between 5 and 50% severe STS (29, 37, 54). In one study, a mediation analysis was conducted for STS, and a secure attachment style was found to partially mediate the effect of primary traumatization on STS (29). A non-significant trend for women having higher STS symptoms than men was found (29), although no such differences were detected in another interpreter sample (37). STS showed a significant negative correlation with social support and sense of coherence, and a significant positive correlation with a preoccupied attachment style (29). No significant correlations emerged for work experience, flight experience, or employment (29). Wichmann et al. (37) also examined several factors regarding STS and challenges in a triadic setting, e.g., first-person interpreting or feeling equal in the triad, but no significant findings emerged. Moreover, no significant results were reported for vicarious or secondary traumatization (53, 55).

Burnout was investigated in two studies (53, 55). Interpreters who had experienced trauma in the past or received supervision showed significantly higher burnout levels than did interpreters without trauma or supervision (55). The author of the latter study suggested that supervision might not have addressed the interpreters' issues and was hence not efficient in reducing stress. Additionally, interpreters who underwent supervision might have also experienced more work-related stress, e.g., because of higher workload or more complex client. Work experience (number of months working as an interpreter) and workload (weekly hours) were significantly positively correlated with burnout (55). CF and burnout symptoms were low for interpreters compared to the norm of the applied questionnaire, and therapists had significantly higher compassion fatigue and burnout levels than did interpreters (53).

##### Positive Emotional Reactions and Motivations

Interpreters reported various positive reactions which were related to the concept of CS (*k* = 7), such as feeling rewarded (41) or doing a meaningful job (42). Moreover, they described a healing process through interpreting regarding their own war-related distress (*k* = 6). Some interpreters picked up coping strategies (50) or techniques such as sleep hygiene (43). Several interpreters explained their motivation for interpreting (*k* = 8), including achieving a professional status (44, 52) or working with people (2, 39, 52). Other motivations were rather personal, such as helping one's countrymen (12, 44, 52), feeling responsible (48, 51), and personal reasons such as helping themselves (12, 43, 44, 52).

In a quantitative study, all interpreters experienced their work as rather or very meaningful (53). Compassion satisfaction was examined in two studies (53, 55), and showed a significant negative correlation with the number of hours spent working with traumatised clients per week (55). One possible reason for this finding could be that these interpreters experienced high levels of stress, but by working fewer hours they reached a level where they were still satisfied with their work. In one study, interpreters reported the motivation for their work as follows: wish to help (57%), financial reasons (27%), personal or professional development (13%), empathy with refugee clients (13%), and being an interpreter [12%, (37)].

##### Coping Strategies

Interpreters mentioned several adaptive coping strategies, e.g., distracting activities (*k* = 5) or emotion regulation (*k* = 6). Cognitive strategies (*k* = 9) included distancing through professional explanations (38) or writing clients' stories down (38, 43). Some interpreters sought social support from family, friends (41, 43), health care staff (41, 44), and peers (41, 43, 50). However, maladaptive strategies were mentioned as well as some interpreters were overwhelmed by their job and started to avoid assignments in the mental health setting (41, 51). In three studies, interpreters reported to have quit their work as interpreters (12, 36, 40).

In quantitative studies, social support in terms of talking to friends, family, or health care staff (37, 56) was also mentioned as an adaptive coping strategy. Further adaptive coping strategies included hobbies and sports (37, 56), religion (56), seeking professional help (37, 56), separation of private and work life (37), and a sense of meaningfulness at work (37). In one study, 35% of the interpreters indicated to have sought psychological help at some point in their life (37). At the time of the survey, 13% indicated to currently receive psychological treatment and 13% expressed a wish for treatment (37). However, maladaptive coping strategies were mentioned as well such as rumination (56), and alcohol consumption (37, 56).

#### Working in a Triad

The second third-order theme, “Working in a triad,” consists of three second-order themes (relationship to client, relationship to professional, and role in triad) and 27 first-order themes (Table 4). It covers problematic situations and dynamics regarding the relationship to the client, to the practitioner, and the role in the triad. These experiences were often related to the interpreters' emotions and satisfaction with their work. The second-order theme “relationship to client” highlights the interpreters' conflicts, which were often associated with keeping a distance from the client and uncertainties about how to shape their relationship with the client. The second-order theme “relationship to practitioner” is primarily characterised by negative experiences with practitioners, which seemed to affect interpreters' job satisfaction. The second-order theme “role in triad” describes the frequent contradictions between the various role expectations faced by interpreters and their own intentions and perspectives. Only one quantitative study contributed to this third-order theme (37), and provided information particularly about the dynamics in the triad.

**Table 4:** Working in a triad as third-order theme specified by first-order and second-order themes.

**Second-order theme**	**First-order theme**	**References**
Relationship to client	Strong sense of empathy	(20, 39, 40, 44, 47, 48, 50, 51)
	Shared experiences/origin helpful and challenging	(12, 36, 38, 41, 43, 48–52)
	Ambivalent feelings towards client	(2, 41, 42, 46, 48, 51, 52)
	Trying to be neutral to client	(2, 41, 42)
	Keeping distance from client	(20, 43, 49, 51)
	Acting informal is helpful	(39, 48–50)
	Trust is important and how to create it	(2, 20, 39, 42, 45, 48, 49, 51)
	Disrespectful clients	(45, 51, 52)
	Clients' wrong expectations of interpreters	(2, 20, 42, 48, 49, 51, 52)
Relationship to practitioner	Feeling disrespected by practitioner	(12, 36, 42–44, 51, 52)
	Concerned about practitioner's attitude	(41, 46, 48, 51, 52)
	Feeling powerless	(44, 48, 52)
	Positive and productive relationship	(12, 20, 42, 46–49, 51)
	Staff's limited understanding of interpreter work	(36, 41, 43, 52)
Role in triad	Sensitive position in triad	(12, 20, 42, 47, 49, 51, 52)
	Interpreter's role interferes with motivation to help	(2, 20, 41–43, 45, 48, 49, 51, 52)
	Doing more than interpreting	(2, 41, 44, 46, 51, 52)
	Feeling restricted	(20, 43, 48)
	Valuing a neutral role	(20, 42, 43, 45)
	Impartiality important but challenging	(20, 42, 43, 49, 50, 52)
	Dilemma between showing and containing emotions	(36, 40, 41, 43, 48, 50)
	Handling various expectations	(42, 43, 50–52)
	Interpreting demands	(12, 20, 36, 38, 41–43, 47–49, 51)
	Being an active part in therapy	(20, 39, 42, 43, 47, 48)
	Triad as positive workplace	(41, 42, 46, 48, 49)
	Experience and therapy as resources	(12, 48, 52)
	What is an ideal interpreter	(20, 36, 38, 45, 47, 48, 50)

##### Relationship to Client

Interpreters reported ambivalent feelings and motivations regarding the client (*k* = 7), e.g., strong sympathy (41, 48) and hospitality (46), along with feeling burdened (48). Moreover, some interpreters faced negative attitudes (*k* = 3) and high expectations of clients (*k* = 7), e.g., because clients thought that the interpreter was the decision-maker (52) or had high expectations because the interpreter was from the same country (49). Shared origin and/or experiences resulted in advantages and disadvantages for the interpreter (*k* = 10). On the one hand, it helped to develop a productive working relationship (38, 49, 51, 52), as interpreters understood clients more easily (49) or the shared cultural background helped them to be an adequate interpreter (52). On the other hand, interpreters also felt reminded of their own past trauma in a stressful way (12, 41, 43, 48).

Trust emerged as a separate theme (*k* = 8), with interpreters emphasising the importance of trust within the triad, especially with refugee clients (2). They elaborated that creating trust needed time (45, 51) and sometimes developed first between the client and the interpreter (49) and then extended to the practitioner (51). Interpreters mentioned various factors as trust-enhancing and comforting for the client, such as body language (2, 20, 42), the shared culture and history (42, 48, 51), knowing about the client's trauma history (2), transparency (42), and saying positive things about the practitioner (51).

In a quantitative study, interpreters mentioned several challenging aspects regarding the client, such as culturally caused conflicts (17%), culturally caused taboos (22%), mistrust towards the practitioner (27%), and mistrust towards the interpreter (7%) (37).

##### Relationship to Practitioner

Most of the first-order themes referred to negative experiences with practitioners such as “feeling disrespected by practitioner,” “staff's limited understanding of interpreter work,” or “feeling powerless.” Negative experiences with the practitioners (e.g., psychotherapist, clinician) were for example: feeling powerless, low level of respect (12, 42, 43, 51), and feeling like an object (12, 44, 51). Many practitioners were perceived as misunderstanding the interpreter's job (36, 41, 43, 52). Nevertheless, interpreters described positive and cooperative relationships too, in which they were asked for their opinions and ideas (20, 42).

Interpreters reported several challenging aspects regarding the practitioner in a quantitative study, e.g., worry that the therapeutic relationship could be weakened (18%), fear of mistranslations (22%), lack of knowledge/understanding of cultural particularities (33%), and lack of knowledge/understanding of the client's social and socioeconomic background (22%) (37).

##### Role in Triad

The role in the triad with practitioners and clients was found to be versatile. Translating between practitioner and client was described as being of a sensitive nature (12, 20, 42, 47, 51), as interpreters wanted to help without crossing professional boundaries (20, 47). However, interpreters mentioned several activities which went beyond the interpreter's role in the session (*k* = 6), e.g., practical help with authorities (46) or advocating that the client leaves the practitioner (51). Acting as a conduit was either perceived as safe as they preferred not to be involved to much (43, 45) or as restricting as some interpreters wanted to do more than translating to help clients (43, 48). Some saw their active role positively (20, 42, 48), while others felt it to be a burden (42). Impartiality was perceived as important (20, 42, 49, 50, 52) but demanding (43, 50) and conflicting with cultural values (43).

Interpreters described several positive feelings within the triad (41, 42, 46), e.g., feeling appreciated (41) or accepted (46). However, the interpreter's work was also perceived as demanding (12, 38, 41, 43). For example, cultural misunderstandings and difficulties were mentioned as stressful (12, 49), as were problems regarding the translation process, e.g., clients mumbling, or too long sentences (36).

In various studies, interpreters' thoughts about how they should act appeared to be idealistic (*k* = 7), e.g., being able to cope with everything (36), having excellent knowledge about the client's culture and language, positive interpersonal attitude, good communication skills (20), containing one's own emotions (38, 45, 50).

In the quantitative study, 82% of the interpreters regarded themselves as language and cultural brokers, 52% saw themselves as advocates or helpers, and 32% identified only as interpreters. They also reported several aspects which helped to maintain neutrality, such as emotional/social/mental distance (23%) or work experience or education (12%). Several interpreters supported clients in other ways than translating, e.g., assistance in dealing with authorities (28%), translating documents (28%), help in filling out documents (23%), invitations (e.g., to private parties) (12%). Overall, interpreters received information about the working conditions (57%). and experienced a clear distribution of responsibilities (98%), personal recognition by practitioners (93%), professional recognition by practitioners (97%), and equality in the cooperation with practitioners (85%). Moreover, the majority reported that practitioners clarified misunderstandings (83%), responded to difficulties encountered (72%), and provided feedback (72%). The importance of trained practitioners for work with interpreters was emphasised by 85% of the sample. In this study, 93% considered the cooperation as medium, good or very good (37).

#### Working Environment

The final third-order theme “Working environment” deals with various organisational aspects of the work and their consequences for the interpreters' professional and personal well-being. It is divided into two second-order themes: “working conditions” and “suggestions for improvement” (Table 5). In this regard, interpreters described and complained about working conditions that often revealed deficits in the support system and stressful circumstances with which they had to deal. In addition, various suggestions for improvement were made regarding the working conditions of interpreters.

**Table 5:** Working environment as third-order theme specified by first-order and second-order themes.

**Second-order theme**	**First-order theme**	**References**
Working conditions	Mental health setting is special	(12, 20, 41, 43, 47, 48, 50)
	Clients require special attention	(2, 20, 41, 51, 52)
	Having to get to know Western therapy	(42, 48, 51)
	More trust in community interpreter than agency interpreter	(39, 42, 52)
	Working in own community challenging	(2, 49, 51, 52)
	Confidentiality important but difficult	(20, 41, 42, 44, 48)
	Organisational difficulties	(12, 20, 38, 40, 41, 45, 49, 51)
	Noticing racism towards client	(51, 52)
	Often lack of recognition of staff and agency	(12, 20, 43, 44, 48, 51, 52)
	Poor remuneration	(12, 41)
	No preparation	(12, 40, 43, 44)
	Rare briefing	(12, 36, 41, 49)
	Debriefing important but rare	(12, 36, 44, 45, 48, 50, 51)
	Often lack of supervision	(12, 36, 50)
	Possibility to get support	(12, 36, 38, 44–46, 48, 50, 51)
	No training, training is important, no training can prepare, training is not sufficient	(12, 36, 44, 48, 50, 51)
Suggestions for improvement	Briefing	(2, 36, 40, 41, 43, 49)
	Debriefing	(36, 40, 41)
	Training	(12, 36, 40, 41, 45)
	Professional support	(12, 36, 44)
	More awareness by and training for professionals	(12, 36, 41, 51)
	Work setting	(12, 40, 41, 43, 50)

##### Working Conditions

The mental health setting was perceived as unique (47), demanding (12, 43), and requiring more attention compared to other work settings (20, 52). The concept of therapy was not always known or clear to interpreters (42, 48, 51).

Various types of potential support by employers were mentioned. Interpreters reported possibilities for briefing (*k* = 4), debriefing (*k* = 7), training (*k* = 6), and supervision (*k* = 3). Interestingly, some described access to training to be vital (51), whereas others believed that no amount of training would adequately prepare them for appointments with refugee clients (36), or stated that the training obtained lacked certain aspects like interpretation ethics or techniques, e.g., translating everything or maintaining confidentiality in support groups (12, 44, 48). Likewise, it was suggested that the specific characteristics of mental health settings should be included in training programmes for interpreters, as they are intensely confronted with traumatic content (45). In several studies, interpreters reported devaluing experiences regarding the service with which they worked (12, 43, 44, 48, 52). Interpreters felt pressured (12), treated as second-rate employees (44), or abused by the service with which they worked (48). Payment was perceived as poor (12, 41), and external conditions, like inattentive clients (41) and lack of time and breaks (12, 38), were perceived as hindering for the interpreter job. Racism towards the clients was also observed (51, 52), and negatively affected the interpreters (51).

Interpreters reported actively seeking support from their employers (36, 45, 48, 50) and peers (12). Reasons for not accessing or seeking support were no offer by the employer (36, 38), strict confidentiality rules (12, 44), financial and time pressures or feeling unworthy of support (48), and no knowledge to whom to turn to for support (36, 51). Whereas, some interpreters tried to deal with their work without support (36), others expressed that interpreters would not need such support as they were mentally strong (48, 51).

In quantitative studies, 23–38% of the interpreters reported having the opportunity for supervision or case reviews (29, 37). Between 23 and 35% stated having briefings or debriefings (37), 17% had team meetings, and 7% were given the opportunity to undergo training (37).

##### Suggestions for Improvement

Regarding the support from employers, interpreters wished for various offers: training (*k* = 5), briefing (*k* = 6), debriefing (*k* = 3), and counselling or support groups (*k* = 3).

Related to their work setting, interpreters suggested shorter sessions, better coordination or a separate waiting area for the interpreter and the client (12, 40, 41, 43). Interpreters requested that practitioners should also receive training before working with interpreters (12, 36, 51). Moreover, they suggested more sensitivity regarding the interpreter's role (36, 41) and to be made aware of the practitioner's expectations towards the interpreter (41).

In quantitative studies, 22–40% requested debriefing sessions, case reviews, or supervisions (29, 37), and 2% (37) and 41% (29), respectively, wished for training, e.g., to deal with difficult situations (29). Additionally, 20% of the sample would prefer to have additional psychological support (29) or team meetings (37).

### Risk of Bias

The risk of bias rating is displayed separately for qualitative studies (Table 6) and quantitative studies (Table 7). No study was excluded due to risk of bias. However, for several qualitative studies, due to a lack of information, items were rated with “can't tell.” In particular, there was a lack of reporting regarding the recruitment strategy (*k* = 9) and the relationship between participants and researcher in terms of the development of questions and study design (*k* = 12). Measurements were widely appropriate for quantitative studies, but the elaboration of response bias was missing for qualitative studies (*k* = 3). Three studies were declared as pilot studies or small-scale research projects by the authors (12, 44, 52). In two studies, translation and interpreting researchers were involved in the development of the study (36, 39). Therefore, these studies could rather be characterised as assuming a translator's perspective as compared to studies conducted by psychologists, sociologists, or physicians. For example, psychological issues may have been given less weight in studies conducted by translating and interpreting researchers, while there may have been a better understanding of the difficulties and techniques of translation.

**Table 6:** Risk of bias rating for qualitative studies.

**References**	**1. Is a qualitative methodology appropriate?**	**2. Was the recruitment strategy appropriate to the aims of the research?**	**3. Has the relationship between researcher and participants been adequately considered?**	**4. Was the data analysis sufficiently rigorous?**	**5. Is there a clear statement of findings?**
Butler (38)	Yes	Can't tell	Can't tell	Can't tell	No
Celik and Cheesman (39)	Yes	Yes	Yes	Can't tell	Yes
Crezee et al. (36)	Yes	Can't tell	Can't tell	Can't tell	No
d'Ardenne et al. (40)	Yes	Yes	Can't tell	Yes	Can't tell
Doherty et al. (41)	Yes	Yes	Can't tell	Can't tell	No
Dubus (2)	Yes	Can't tell	Can't tell	Yes	Yes
Grant (42)	Yes	Can't tell	Yes	Yes	Yes
Green et al. (43)	Yes	Yes	Can't tell	Yes	Yes
Holmgren et al. (12)	Yes	Yes	Can't tell	Can't tell	No
Lipton et al. (44)	Yes	Can't tell	Can't tell	Can't tell	No
Miller et al. (45)	Yes	Can't tell	Can't tell	Yes	Yes
Mirdal et al. (46)	Yes	Yes	Yes	Yes	Yes
Mirza et al. (47)	Yes	Can't tell	Can't tell	Yes	Yes
Myler (48)	Yes	Yes	Yes	Yes	Can't tell
Resera et al. (20)	yes	can't tell	can't tell	yes	no
Robertson (49)	yes	Yes	yes	yes	yes
Splevins et al. (50)	yes	Yes	yes	yes	yes
Williams (51)	yes	Yes	yes	yes	yes
Williams (52)	yes	can't tell	can't tell	can't tell	no

**Table 7:** Risk of bias rating for quantitative studies.

**References**	**1. Is the sampling strategy relevant to address the research question?**	**2. Is the sample representative of the target population?**	**3. Are the measurements appropriate?**	**4. Is the risk of non-response bias low?**
Birck (53)	Can't tell	Yes	Yes	Yes
Denkinger et al. (54)	Yes	Yes	Yes	Yes
Kindermann et al. (29)	Can't tell	Can't tell	Yes	Yes
Shlesinger (55)	Can't tell	Yes	Yes	No
Teegen and Gönnenwein (56)	Can't tell	Can't tell	Yes	No
Wichmann et al. (37)	Yes	Yes	Can't tell	No

## Discussion

This systematic review examined mental health and work experiences of interpreters working in mental health care settings for refugees. Overall, 19 qualitative studies and six quantitative studies were identified. The sample sizes varied extensively between the studies (ranging from 3 to 90), with several qualitative studies having only three to five participants. In total, a third of the included studies were conducted in a mixed setting that did not specifically focus on experiences in the mental health setting. Therefore, work settings were very heterogeneous. A thematic analysis was applied to identify themes in qualitative studies. The emerging themes were similar to those found in a review focussing on interpreters' experiences in health and mental health settings (10). The three superordinate themes of the thematic analysis were “Emotions, behaviour, and coping strategies,” “Working in a Triad,” and “Working Environment.” In general, the results rely on mostly qualitative studies with small sample sizes and less on quantitative studies with heterogeneous samples with exception of one second-order theme (“negative emotional reactions and behaviour”). Here, a strong imbalance becomes apparent in terms of the contribution of qualitative and quantitative studies to the different themes, as most quantitative studies focused on mental health. At the same time, there are very few quantitative studies so far, so the quantitative results have been broken down as precisely as possible and this second-order theme includes particularly many results of quantitative studies. Qualitative studies provided an insight into the different emotions felt by interpreters during and after their work. Moreover, the interpreter's position in the triad appeared complex and was perceived in multiple ways. It was not possible to carry out a meta-analysis as almost every quantitative study focussed on a different measurement of psychological strain. Overall, interpreters showed elevated stress and anxiety levels in a quantitative study (29). Also, PTSD prevalence was higher in interpreters (29, 56) than in a representative sample of the German population [2.3%, (58)], and STS was investigated most extensively in quantitative studies.

### Illustration of a Model of Risk and Protective Factors of Interpreters

**Figure 3:**
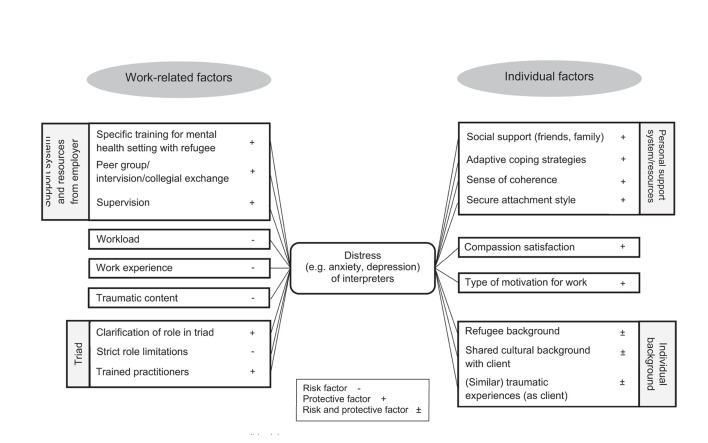
Illustration of a theoretical model for possible risk and protective factors of interpreters' distress.

Most of the quantitative and qualitative results rely on singular studies with heterogeneous samples. However, they might give directions for risk and protective factors regarding the distress among interpreters. Therefore, a theoretical model is presented in Figure 3, which summarises the qualitative and quantitative results and assigns them to possible risk and protective factors for distress of interpreters. Some factors are presented as both protective and risk factors, because the results were ambiguous (e.g., refugee background was not associated with STS in a quantitative study whereas some reports in qualitative studies indicated that having a refugee background was associated with difficult emotions while interpreting).

The qualitative and descriptive quantitative results regarding “negative emotional and behavioural reactions” (second-order theme) are summarised as distress in the middle of the model. However, the first-order theme “traumatic content stressful” of this second-order theme is represented as risk factor (left-hand side: “traumatic content”) as it was associated with distress in the results. The risk and protective factors are divided into work-related and individual-related factors. The results of the third-order themes “Working conditions” and “Working in a triad” are summarised on the left-hand side of the figure as part of the work-related factors, because these themes consider the dynamics and conditions in the interpreter's work situation. Only one first-order theme (“shared experiences/origin helpful and challenging”) of “Working in a triad” is presented as a single factor within “individual factors” on the right-hand side. Individual factors were therefore derived from the second-order themes positive emotional reactions and motivations and coping strategies.

Some of the included risk and protective factors were shown to be correlated with psychological strain (e.g., workload, work experience). Other factors were investigated on a descriptive level in quantitative studies and were related to psychological strain in qualitative studies (e.g., functional coping strategies). In the following sections, we discuss the role of the postulated risk and protective factors in greater detail.

#### Support System and Resources From Employer

The quantitative and qualitative results of the present review indicate that interpreters are longing for supervision, but rarely have access to it. Supervisions as suggested for trauma-informed practise could include topics such as helping interpreters structure their workload or developing cognitive strategies to better separate their work with refugee traumatised clients from their private lives (59). In terms of seeking support, the results of the present review are similar to those reported in a Australian study with public service interpreters (6) that was not included in the review as clients and setting were not specified. In this study, a lack of knowledge about where to get support, a lack of money, or misconceptions about professionalism as well as a lack of role models for interpreters appeared to be barriers to seeking support. Therefore, a support system (e.g., supervision) from the employer was classified as a protective factor in the model. The concept of supervision might also be unfamiliar to some interpreters and should be introduced before beginning the job. Additionally, in order to investigate the interpreters' job satisfaction and burnout, the quality of supervision should be adequately evaluated and taken into account. In general, the lack of recognition, support, and organisation might also contribute to a higher degree of burnout, distress, and frustration, but these associations have yet to be investigated.

#### Work Experience, Workload, and Traumatic Content

Work-related factors such as experience and workload showed positive (55) as well no correlations (29, 37) with psychological strain. Little is known about whether interpreters work full-time or part-time. In one study though, almost 50% worked part-time (37). A high workload and/or low professional experience could therefore contribute to higher levels of distress and thus be risk factors for the interpreter's well-being. However, as all of the included studies were cross-sectional, there is no evidence about emotional stress over time. It remains to be investigated whether work experience serves as buffer against psychological stress and/or whether acquired coping strategies might help, as qualitative studies suggested. Additionally, interpreters might also report stress at a specific time, e.g., when they accompany severely traumatised clients. The qualitative results also indicate an impact of traumatic content on the interpreter's distress. One study in the present review explored the relation of STS of interpreters with the caseload of traumatised clients and with clients who talk about their trauma, and revealed no significant findings (37). Hence, future studies should consider work stressors (e.g., traumatic content, workload, possibility to take breaks) in the interpreter's work when investigating mental health, especially regarding freelance interpreters. Overall, the results suggest that guidelines on how to work with interpreters in refugee settings would be helpful in order to improve the situation for interpreters, e.g., by offering paid supervision or ensure break time between sessions.

#### Triad

Several aspects regarding the role and relationships within the triad emerged as themes in the qualitative analysis but were barely explored in quantitative studies. The perceived discrepancy between the formally assigned role by the mental health practitioners and the actually executed role of interpreters is a widely discussed topic (4, 5). On the interpreter's side, the results of the review suggest an ambiguity regarding the interpreters' preferred and actual involvement in the process of the therapeutic session. This confirms the findings of a previous review exploring interpreter roles in the clinical setting (5), which identified roles such as cultural broker, clarifier, or patient advocate. In various guidelines, it is outlined that contact between client and interpreters outside sessions should be limited (9) and private contacts are not allowed (19). At this point, a general clarification of the interpreter's role does not yet appear to have been accomplished in terms of global or even national guidelines. This is due to the various employment situations and different degrees of education and training of interpreters. However, in light of the present review findings, a clarification of the role within the triad could serve as a protective factor for the interpreter's well-being. There is therefore an urgent need to develop a general job description that defines tasks and responsibilities for interpreters. This could then be written into contracts and job descriptions to create more transparency for all involved. Importantly, this should be developed and discussed jointly between practitioners and interpreters in order to avoid misunderstandings and to include both perspectives. Additionally, there appear to be no networks for interpreters working with refugees in this field. As there is still little or no lobby for interpreters, their need for support remains poorly addressed and sometimes, as in Germany there is no legal entitlement for professional interpreters. Networks could help bring together mental health experts such as policy makers, practitioners, and interpreters to develop new structures that provide a better working environment for interpreters and a more effective care for refugee clients.

The thematic analysis also revealed that interpreters often perceive themselves to be a technical tool or feel restricted because some interpreters wanted to give more advice, be more involved in the therapy process. Furthermore, they also described this role as stressful or conflictual. Too strict role limitations could therefore be a risk factor. Hence, practitioners should set clear role expectations before sessions and be open to regular exchange between practitioner and interpreter. In preparatory trainings, interpreters could furthermore be advised on how to behave when they feel they want to intervene and when this is allowed. This might improve the interpreter's perceived safety and enhance the relationship between practitioner and interpreter.

Qualitative review findings showed that from the interpreters' perspective, it appeared that practitioners often do not understand the interpreters' role and have different expectations. As stated in a guideline article (9), practitioners rarely receive training in working with interpreters, even though this is recommended. A recent scoping review moreover strongly emphasised that interpreter and practitioner should form a cooperative relationship and can even be trained and supervised together (26). Thus, working with trained practitioners could be a protective factor. Generally, the present results reinforce the idea of a professional team in which both sides are trained regarding the work with one another.

#### Personal Support System/Resources

In particular, social support, e.g., from family and friends, and functional coping strategies such as sports or hobbies, were described as a personal resource in several qualitative and quantitative studies. A sense of coherence and a secure attachment style were derived as protective factors. Kindermann et al. (29) suggested that interpreters may benefit from trainings with a focus on sense of coherence as they were proved to be helpful for health care workers in rescue services. Additionally, they suggested that feedback and supervision may be helpful for interpreters for insecure attachment style. However, as those factors are based on the results of a single included study, they need to be interpreted with caution. In general, there is little research on interpreters' personal resources. However, research on personal resources could contribute to a better understanding of stress and be helpful in developing preparatory training for interpreters.

#### Compassion Satisfaction and Type of Motivation for Work

None of the included quantitative studies investigated CS in relation to psychological strain, although several studies qualitatively reported experiences similar to CS, e.g., valuing work or doing a meaningful job. The qualitative reports indicate a strong positive association between CS and interpreters' well-being, for example when interpreters felt satisfied or stimulated by their work. Also, research with interpreters for LEP clients shows a negative correlation between CS and burnout (16). In addition, relations between interpreters' job motivation and distress have not yet been examined, but could act a possible correlate for interpreters' mental health based on the presented qualitative findings. For example, interpreters who pursue the profession because they want to help people from similar cultural backgrounds may experience stress differently than those who pursue the profession for financial reasons. In some reports, motivation was clearly linked to well-being, e.g., satisfying a need for belonging, regaining self-confidence or working to reduce one's frustration (12, 44). Overall, this may indicate that motivations can have an impact on distress. Future studies should therefore consider the relationships between CS and motivation with distress and job satisfaction, as these may act as protective factors.

#### Individual Background

The relation between trauma exposure on the part of the interpreter and work-related psychological strain was primarily pointed out in qualitative studies. Only one quantitative study examined trauma exposure of the interpreter, but the respective information was very limited (i.e., participants were merely asked whether they had ever experienced a trauma). Although shared experiences were perceived differently, the advantage of shared experiences with the client was emphasised several times by the interpreters. Therefore, trauma exposure, especially war- and flight-related, might even mitigate negative emotions related to interpreted traumatic content if the interpreter has processed his/her trauma successfully. Sharing the same or a similar cultural background might also positively influence the work in the triad. However, in a study that was excluded as the clients were not specified, STS, burnout, and CS of the interpreter were not linked to a refugee status of the interpreters (16). By contrast, in a study with refugees (who were not interpreters), the number of experienced trauma and post-migration stressors like loss of culture and support were reported as significant correlates for emotional stress (60). As the association between post-migration factors and psychopathology is a common finding in refugee populations, it is to be expected that this is also the case with interpreters who were refugees themselves. Therefore, all factors of the individual background are presented as both risk and protective factors. Additionally, flight-associated variables such as post-migration stressors and trauma load might be more relevant as possible risk and protective factors for interpreters' distress, as they reflect more specific overlaps with the client's case. Therefore, interpreters in particular, who have had similar traumatic experiences as the refugee clients, should be prepared in advance for potentially traumatic content and ways of coping.

#### Evaluation of the Model of Risk and Protective Factors

Overall, little is known about risk and protective factors regarding interpreters' general and work-related mental health. The presented model might therefore give an overview of relevant aspects for working as and with interpreters. However, most factors refer to a single quantitative outcome in particular studies which differ from each other in methodological terms. Some factors are based almost exclusively on qualitative studies, but these studies often have very small and specific samples. Therefore, the association between mental health and the proposed factors has to be interpreted with caution because the individual components in this model are equally weighted, even though they are based on different numbers of participant reports. Future studies should investigate the proposed risk and protective factors in order to gain a better understanding of their individual impact on interpreters' mental health. Nevertheless, this is the first model that summarises risk and protection factors based on a systematic review and can therefore provide implications for improved work with refugee clients in mental health care.

## Limitations

While the presented systematic review focussed on mental health settings, one third of the included studies did not exclusively refer to such settings. Nevertheless, among the qualitative studies, more studies from the mental health setting contributed to first-order themes than mixed-settings studies. Two quantitative surveys (37, 56) recruited interpreters primarily from psychosocial treatment centres, where mostly psychological support was provided. Therefore, it can be assumed that the majority of the interpreters in these studies worked in mental health care and associated their experiences with the mental health setting.

All studies had a cross-sectional design with convenience samples. The proposed risk and protective factors only provide hints as to how the interpreters' distress might be influenced, and should, for the time being, be seen as correlates in rather specific cases. Moreover, qualitative studies in particular showed small sample sizes and often had heterogeneous participants. Most of the results cannot be related to specific work environments and sociodemographic characteristics of interpreters, as these were often not reported. Therefore, the qualitative results can only be interpreted and generalised with great caution. Additionally, the risk of bias rating showed a lack of information regarding the study design in several studies. Hence, it is often not clear how participants were recruited and how the researchers' role affected the study process. The quantitative results are generally very scarce and include heterogeneous work settings (e.g., mental health care and court combined), samples (voluntary and paid interpreters), and measurements (e.g., stress, anxiety). In general, results for a specific outcome were available only in single samples. Therefore, a meta-analysis was not possible due to outcome and sample heterogeneity. Moreover, all of the studies were carried out in Western countries, and are therefore likely to represent a Westernised system of interpreting work and health care systems. Five of the six quantitative studies were conducted in Germany, and their findings might therefore also be influenced by the characteristics of the German health system.

Depending on the country and setting, paid interpreters are sent by agencies, the respective health service in the country under study or are employed by humanitarian organisations (25). Samples in the included studies were recruited in NGOs, hospitals, or through anonymous surveys. Therefore, interpreters had different employment conditions (e.g., freelancer or employed interpreter) and were probably subject to various different policies, rules, and instructions. In consequence, interpreters might have expressed concerns relating to their own very specific work situation. Overall, however, the results suggest shortcomings in all of the different work environments.

## Conclusions and Implications

To the best of our knowledge, this is the first systematic review to examine and summarise qualitative and quantitative studies on mental health and related work experiences of interpreters working in the mental health setting with refugee clients. The two different methodological approaches allowed us to identify and compare quantitative and qualitative results. Although all studies included in this review applied only cross-sectional designs, it can be assumed that interpreters are highly affected by their work. A model of possible risk and protective factors is presented. However, several of the presented factors are based on results of individual studies and must therefore be interpreted with caution.

Various factors associated with the work environment, such as payment, recognition, and support by employers, have not yet been investigated with a quantitative approach, which should therefore be considered in future studies. Interpreters still appear to be unseen by practitioners, and the constant wish for more support suggests a gap between published policies and current practise. Moreover, the varying employment conditions give rise to a complex situation, in which it remains to be clarified who has to provide support and training depending on the employment situation.

More quantitative research is needed regarding interpreters' experiences specifically in the mental health setting. All included studies were published in the last two decades, which emphasises the increasing importance of the interpreter's situation and mental health. The overall analysis also reveals an increased psychological strain in interpreters, not only in the mental health setting but in diverse settings. Future studies should therefore focus on interpreters' mental health related to specific work settings. Moreover, identifying potential protective and risk factors will improve the development of treatment and care for refugees in the specific settings.

## Data Availability Statement

The original contributions presented in the study are included in the article/Supplementary Material, further inquiries can be directed to the corresponding author.

## Author Contributions

AG and NS designed the systematic review and developed the search strategy. AG coordinated the literature research, the literature screening, the risk of bias rating, performed the data collection for quantitative and qualitative data, and drafted the manuscript. NS supervised the systematic review. NS, MB, and CK revised the manuscript. All authors contributed to the article and approved the submitted version.

## Funding

The Stiftung der deutschen Wirtschaft provided doctoral funding for AG. The funding source was not involved in the design, implementation, analysis, or reporting of the results.

## Conflict of Interest

The authors declare that the research was conducted in the absence of any commercial or financial relationships that could be construed as a potential conflict of interest.

## Publisher's Note

All claims expressed in this article are solely those of the authors and do not necessarily represent those of their affiliated organizations, or those of the publisher, the editors and the reviewers. Any product that may be evaluated in this article, or claim that may be made by its manufacturer, is not guaranteed or endorsed by the publisher.
