# Supplementary material for: Mental Health and Work Experiences of Interpreters in the Mental Health Care of Refugees: A Systematic Review
Source: Front Psychiatry. 2021 Oct 18;12:710789. doi: 10.3389/fpsyt.2021.710789 (PMC8558553; doi:10.3389/fpsyt.2021.710789)
Supplement: Supplementary file 1 [file Table_1.DOCX]

Supplementary Material

Questionnaires applied in quantitative studies

| **Reference** | **questionnaire** | **authors (year)** | **construct** |
| --- | --- | --- | --- |
|  |  |  |  |
| Birck (2001) | Traumatic Stress Institute Belief Scale | Pearlman (2000) | Disruptions in the areas of safety, trust, esteem, intimacy and control |
|  | Compassion Satisfaction/Fatigue Self Test | Stamm (2000) | Compassion fatigue and satisfaction, burnout |
| Denkinger et al. (2018) | Questionnaire for Secondary Traumatization (FST) | Daniels (2006) | Secondary traumatic stress |
|  | Relationship Questionnaire | O’Connor, Elklit (2008) | Adult attachment styles |
|  | distressing factors and resources | developed for reported study | Distressing factors: e.g., Reports on beneficiaries’ traumatic experiences, Witnessing the suffering of beneficiaries  Resources: e.g., Appreciation from beneficiaries, Supervision |
| Kindermann et al. (2017) | Essen Trauma Inventory | Tagay, Stoelk, Möllering, Erim, Senf (2004) | Trauma exposure and PTSD symptoms and diagnosis |
|  | Questionnaire for Secondary Traumatization (FST) | Daniels (2006) | Secondary traumatic stress |
|  | depression module of the Patient Health Questionnaire (PHQ): (PHQ-9) | Löwe, Spitzer, Zipfel, Herzog (2002) | Severity of depressive symptoms |
|  | Anxiety module of the PHQ: (GAD-7) | Löwe, Decker, Müller, Brähler, Schellberg, Herzog, Herzberg (2008) | Symptoms of generalised anxiety |
|  | Perceived Stress Scale (PSS-10) | Cohen, Kamarck, Mermelstein (1983) | Perceived stress level |
|  | Sense of Coherence Scale (SOC-29) | Abel, Kohlmann, Noack, Noack (1995) | Sense of coherence |
|  | Social Support Questionnaire (F-SozU K-14) | Fydrich, Sommer, Brähler (2007) | Perceived and anticipated support from one’s environment |
|  | Relationship Questionnaire (RQ) | Asendorpf, Banse, Wilpers, Neyer (1997) | Attachment style |
| Shlesinger (2005) | Professional Quality of Life CSF-R-III | Stamm (2003) | Compassion fatigue and satisfaction |
|  | Trauma and Attachment Belief Scale (TABS) | Pearlman (2003) | Disrupted cognitive schemas |
| Teegen & Gönnenwein (2002) | TLEQ | Zumbeck, Teegen (1997) | Trauma exposure |
|  | PCL-C | Teegen (1997) | PTSD symptoms and diagnosis |
|  | Stress because of interpreter-related job strain | developed for reported study | e.g., Interpreting for emotionally stressed refugees, not being allowed to intervene in the process of therapy or hearing |
|  | Interpreter-related trauma exposure | developed for reported study | e.g., Reports about war-related trauma, rape, torture |
|  | ADS-K | Hautzinger, Bailer (1992) | Depressive symptoms |
|  | FAPK-3 | Koch, 1996 | Scale: emotional communication ability |
|  | Coping strategies for specific endangered occupational groups | Teegen u. a., (1997, 2000) | e.g., Social support, sport, hobbies |
| Wichmann et al. (2018) | Questionnaire for Secondary Traumatization (FST) | Daniels (2006) | Secondary traumatic stress |
